# Real-Time Step Length Estimation in Indoor and Outdoor Scenarios

**DOI:** 10.3390/s22218472

**Published:** 2022-11-03

**Authors:** Zanru Yang, Le Chung Tran, Farzad Safaei, Anh Tuyen Le, Attaphongse Taparugssanagorn

**Affiliations:** 1 School of Electrical, Computer and Telecommunications Engineering, University of Wollongong, Wollongong, NSW 2522, Australia; 2 School of Electrical and Data Engineering, University of Technology Sydney, 15 Broadway, Ultimo, NSW 2007, Australia; 3 School of Engineering and Technology, Asian Institute of Technology, Pathum Thani 12120, Thailand

**Keywords:** step length estimation, RSSI, exponential weighted moving average, path loss

## Abstract

In this paper, human step length is estimated based on the wireless channel properties and the received signal strength indicator (RSSI) method. The path loss between two ankles, called the on-ankle path loss, is converted from the RSSI, which is measured by our developed wearable hardware in indoor and outdoor ambulation scenarios. The human walking step length is estimated by a reliable range of RSSI values. The upper threshold and the lower threshold of this range are determined experimentally. This paper advances our previous step length measurement technique by proposing a novel exponential weighted moving average (EWMA) algorithm to update the upper and lower thresholds, and thus the step length estimation, recursively. The EWMA algorithm allows our measurement technique to process each shorter subset of the dataset, called a time window, and estimate the step length, rather than having to process the whole dataset at a time. The step length is periodically updated on the fly when the time window is “sliding” forwards. Thus, the EWMA algorithm facilitates the step length estimation in real-time. The impact of the EWMA parameter is analysed, and the optimal parameter is discovered for different experimental scenarios. Our experiments show that the EWMA algorithm could achieve comparable accuracy as our previously proposed technique with errors as small as 3.02% and 0.30% for the indoor and outdoor scenarios, respectively, while the processing time required to output an estimation of the step length could be significantly shortened by 53.96% and 60% for the indoor walking and outdoor walking, respectively.

## 1. Introduction

As an essential component in ambulation and gait analysis, the step length appears to be informative in many aspects of life. First and foremost, the step length works as a health indicator, which could aid the diagnosis of injuries and reflect underlying diseases, such as the falling risk [1,2], the prediction of slip severity [3], and the risk of pre-disability [4]. Gait parameters, including step length, step width, cadence, as well as gait speed, can also reflect the fitness of young adults [5]. Moreover, step length and walking speed reflect the mortality and predict the living years of senior citizens to some extent [6,7]. In addition, correctly estimating the step length could contribute to personal localisation, especially in the indoor environment where the Global Positioning System (GPS) is inaccurate or unavailable [8]. Ultimately, step length estimation could also assist the measurement of the social distances between individuals, which could, in turns, help enforce the practice of social distancing [9].

The problem of step length estimation can be traced back to the problem of distance estimation. Meanwhile, stride length is a different, but related parameter, which could be roughly estimated by doubling the step length in a symmetrical walking pattern. Thus, in this section, we review some related works on either step length or stride length measurements. Current gait assessments used by physical therapists are subjective and restrictive, as gait parameters are traditionally observed and recorded by trained professionals and equipment that is costly and not easily accessible [10]. Meanwhile, the testing procedure only lasts for a short period, which could result in inconsistent performance and, thus, may lead to inaccurate conclusions [11]. Thereby, a system or method that can monitor and assess human step length in a continuous daily manner, offering reliable and quantifiable estimation, is worth exploring. In general, step length can be estimated based on three systems, namely the instrumented walkway systems, the vision-based systems, and the wearable device-based systems.

The walkway-based method, such as the instrumented walkway or instrumented treadmill, utilises pressure sensors embedded in mats where participants can get tested while walking on the specifically designed floor covering [12,13,14]. For example, the GAITRite portable mat is a commercially available walkway, which is able to collect data in real-time for instant processing [15]. It relies on embedded pressure sensors to detect the foot positions of the subject under test. This kind of device has been tested reliably in the measurement of the temporal and spatial parameters of gait analysis among both young and old generations [12,16]. The walkway-based measurement is welcomed in certain indoor environments, such as hospitals, clinics, rehabilitation centres, and laboratories, where the sensing mat is laid specially to measure a group of instant gait characteristics and to warn about any abnormal walking behaviours. However, as the walkway system is confined by locations, it is neither cost efficient nor realistic to pave the sensing mat ubiquitously for assessment on a daily basis. Thus, instead of a long-term observation, the sensing mat is only suitable for collecting data in a certain walking session as the participant must walk on the fixed mat in a specific testing premise.

Vision-based monitoring systems have been widely applied around the world. The key feature of vision-based step length detection and estimation is the image processing, which can be performed through cameras and/or infrared radiation (IR) devices. The camera-based system could provide a solution to continuous daily monitoring, which can be used to track individuals’ gait patterns and estimate their step lengths [11,17,18,19,20,21,22,23,24,25,26,27]. A single camera was used in [11] to track the motions of the person under test. The stride length can be estimated by detecting and extracting several pieces of perspective information related to predefined markers. The authors in [28] proposed a two-camera system to extract valuable gait parameters, including the walking speed, step time, and step length. Experimental data were collected from both a laboratory and from senior housing. It was found that subjects performed walking differently in the laboratory and in the senior house. The differences could be as large as 21% in walking speed, 12% in step time, and 6% in step length estimation. Thus, the importance of measuring and estimating the human step length in a real living environment is highlighted. The method of IR thermography was applied in [29] to detect the gait patterns of humans with the estimation errors in the range 9–22%.

Vision-based monitoring systems have several limitations as follows. Firstly, the monitoring cameras or IR devices are required to be installed at a specific place so that the line-of-sight (LoS) path exists, which then limits the horizon of the observation and the range of the movements of the person under test. This is because the participants have to move in a way that the trajectory of the movement is perpendicular to the direction of the camera lens. Measurements in specific, confined locations also have different results compared to those in normal living locations, as shown in [28]. Secondly, their accuracy is affected by obstacles appearing between the cameras or the IR devices and the person under test. Thirdly, the operation of cameras arouses concerns regarding privacy intrusion. Fourthly, because the usage of cameras and IR devices requires well-trained staff and pricey equipment, it is not an ideal method for step length measurements on a daily basis.

The step length estimation using wearable devices is gaining much interest, and the RSSI-based systems (either using RSSI alone or in combination with other approaches such as an inertial measurement unit (IMU)) facilitate the estimation of the step length under different channel models for both indoor and outdoor environments. Step length could be estimated by employing sensors on many body parts. For example, Reference [30] proposed a system that places an IMU sensor on one side of the pelvis. To estimate the step length bilaterally during walking with a single IMU, the authors proposed a novel signalling method by combining a Kalman filter and an optimal filtered direct-and-reverse integration. Results showed that the step length can be estimated for all subjects with errors being less than 3%. Another work [31] focused on long-term monitoring of stride length and gait speed. In [31], a simple geometric model was proposed to estimate the stride length based on the leg length and the opening angle during the stride. To measure this angle, a gyroscope sensor was mounted on the thigh. However, the proposed model had modest accuracy with a ±15% maximum relative error in the estimation of walking velocities. The authors in [32] proposed a novel step detector, which makes use of the opening angle of the leg. It was found that the step length and the opening angle can be formulated with a linear regression model, and the estimated step length error was about 10.37 cm. The research in [33] studied the influence of different factors on the step length and walking speed estimation. By using the Gaussian process regression, with external acceleration to calibrate the measurements, the mean absolute errors for the waist-mounted and ankle-mounted systems were 4.5% and 4.9%, respectively.

The wearable-device-based systems mentioned above have that common drawbacks that the mounting places of the wearable devices have a strong impact on the accuracy of the step length and the RSSI is sensitive to the shadowing effect caused by the human body parts.

In our previous work [34,35], we proposed a step length measurement technique that can overcome the limitations on health-concerning, space-confined, shadowing sensitivity, and daily usage of the aforementioned existing methods. Our technique works based on our developed radio frequency (RF) wearable transceivers, our experimental path loss model, and our proposed data filtering method. The results of our previous work showed that the proposed empirical path loss model between two ankles along with the proposed filtering method in [34,35] could estimate the average human step length with a centimetre error. For example, the step length estimation errors in [34] were only 10.15 mm for indoor walking, 4.40 mm for indoor jogging, 4.81 mm for outdoor walking, and 10.84 mm for outdoor jogging, respectively. However, a limitation of those techniques is that we need to collect the dataset for the whole intended period and then proceed to the offline data processing phase, rather than processing data to estimate the step length and constantly updating this estimation while the person under test is moving. As a result, our previous technique does not facilitate real-time measurements.

Overcoming this limitation is the main motivation of this paper. More specifically, to guarantee both the accuracy and efficiency of the step length estimation and to simulate real-time processing, we propose a novel exponentially weighted moving average (EWMA) technique to continuously estimate the average step length and keep updating this estimation over a shorter period of time. Thereby, the dynamic essence of human activities could be captured more accurately than the simple average used in our previous techniques. The problem at hand is to accurately estimate the human walking step length in real-time so that individuals’ walking patterns could be tracked and updated in a timely manner.

The main contributions of this paper are summarised as follows:

- A novel EWMA algorithm is proposed to determine the threshold pair of the path loss and to update the estimated step length for each segment (or time window) of the dataset. Though the data values are collected offline, the proposed EWMA algorithm provides a perspective to simulate the step length estimation process in real-time.
- The optimal upper threshold for updating the step length estimation for both indoor and outdoor walking scenarios was examined.
- The step length estimation results showed that the accuracy of the proposed EWMA algorithm for indoor walking had a centimetre-level error, which is close to the estimations in our previous works. Interestingly, for outdoor activities, the proposed technique could further minimise the error to only a few millimetres, which is as small as 0.30% of the real step length.
- The processing time of the proposed EWMA algorithm was analysed in comparison with that of our previous work in [34]. Our experiments showed that the proposed EWMA algorithm could save up to 53.96% and 60% of the time required to estimate a step length for the indoor and outdoor walking scenarios, respectively, while it can still provide accuracy comparable to our previous technique.

The rest of this paper is organised as follows. An overview of the moving average algorithms is provided in Section 2. Section 3 presents the experimental setups, including the hardware and software. Section 4 introduces the experimental system model and the proposed EWMA method. The experimental results containing the analysis and evaluation are detailed in Section 5, for both indoor and outdoor environments. Lastly, Section 6 concludes this paper.

## 2. Heuristic Algorithm: Moving Average

The moving average is a favoured tool in statistics for calculating and analysing data by creating a series of averages of different subsets of the full dataset. There are several methods, including simple moving average (SMA), weighted moving average (WMA), and exponential moving average (EMA).

The SMA is the unweighted mean of a numbered data stream. The SMA of a dataset containing *n* points is calculated as

(1) $$SMA=\frac{1}{n}\sum_{i=1}^{n}p_{i},$$

where $p_{i}$ is the *i*-th data point, and every point contributes the same importance to the outcome. SMA was the strategy that we utilised in our previous work [34] to estimate the step length over a dataset. Statistically, SMA is straightforward and relatively accurate when data points do not experience much fluctuation, yet the key limitation is that all data points must be included in the assessment even for those out-of-date ones.

The basic idea of the WMA is as follows. The weighted average is an average that has multiplying factors to give different weights to the data at different positions in the sample time window. Mathematically, it is calculated by multiplying the value of the given data by its associated weighting and totalling the values. In general, WMA assigns a heavier weight to more current data point and less to the past ones. This is because the recent data are more relevant than the data points in the past. The WMA for a time window of *n* data points can be expressed as

(2) $$\begin{array}{cc} WMA & =\frac{q_{n}p_{n}+q_{n-1}p_{n-1}+...+q_{2}p_{2}+q_{1}p_{1}}{\frac{n(n+1)}{2}},\\ \; & =\frac{\sum_{i=1}^{n}q_{i}p_{i}}{\frac{n(n+1)}{2}}, \end{array}$$

where $q_{i}$ is the weight of each data point $p_{i}$, and $\sum_{i=1}^{n}q_{i}=1$.

An EMA is also known as an EWMA. The EWMA for a series *Y* could be calculated recursively as

(3) $$EWMA_{t}=\left\{\begin{array}{cc} Y_{0}, & t=0\\ qEWMA_{t-1}+\left(1-q\right)Y_{t}, & t>0 \end{array}\right.$$

where the coefficient *q* represents the degree of weighting. It is a constant smoothing factor between 0 and 1. The effect of old data samples in Equation (3) reduces exponentially fast. A smaller *q* discounts the past observations faster. $Y_{t}$ is the value at a time period *t*, and $EWMA_{t}$ is the value of the exponential weighted moving average at the time period *t*.

Though the basic smoothing provided by the SMA may be effective for a finite number of data, it is concerning that the effects of lags in the data may reduce the responsiveness of the moving average indicator. The WMA and EWMA are more responsive to the change of data as they rely more heavily on the freshest data and place less value on older ones. This suggests that the EWMA might be a better approach to estimate the step length in a daily human activity. As a result, this paper proposes a novel EWMA algorithm to estimate the step length in both indoor and outdoor walking scenarios. It is worth noting that the pure EWMA technique already exists, but has not been used in step length estimations. Thus, our paper is the first work that uses the EWMA technique to estimate step length. In addition, apart from updating the mean value μ and the standard deviation value σ in each time window as in the pure EWMA technique, the upper threshold of our data filtering technique is found to be in the form of μ+γσ. To estimate the human step length accurately, the optimal value of γ also needs to be explored. The whole process of estimating μ, σ, γ, and thus, the upper threshold of the corresponding time window is called our novel EWMA algorithm.

## 3. Experimental Setup

In this paper, the experiments were carried out by deploying the same hardware, as well as software and under the same environments as in [34,35]. This means that two Arduino UNO microprocessors were programmed by the Arduino Integrated Development Environment (IDE) as a transmitter (coordinator) and a receiver (end device), respectively. XBee-PRO S2C wireless module chips were configured by the XBee Configuration & Test Unit (X-CTU) software, including destination and source addresses, interface data rate, and power level. A signal will be transmitted with the power of $P_{0}=0$ dBm, at a rate of 9600 bps, from the coordinator to the end device. The operating frequency of the RF modules is 2.4 GHz in the Industrial, Scientific, and Medical (ISM) band [36], which is also one of the carrier frequencies of the IEEE standard recommended for wireless body area networks [37]. The data samples are received and stored in a micro SD card, then imported to and analysed in Matlab.

**Figure 1:**
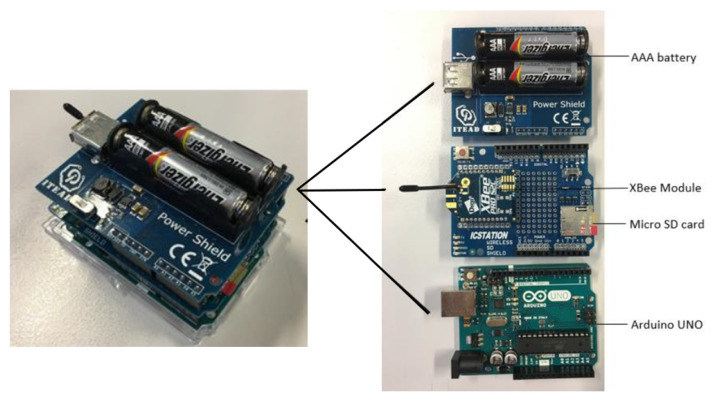
Components of the transceivers.

**Figure 2:**
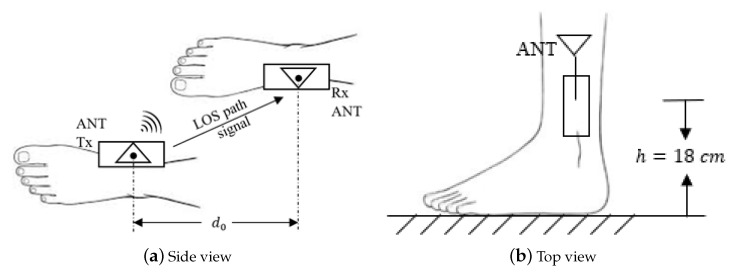
Schematic diagrams of the on-ankle transceivers.

After assembling the transceiver hardware, whose components are shown in Figure 1, the equipment will be attached to the medial side of human ankles at the same height *h*, as illustrated in Figure 2. The distance between the two antennas of the transceivers in Figure 2 is defined as the real step length $d_{0}$.

**Figure 3:**
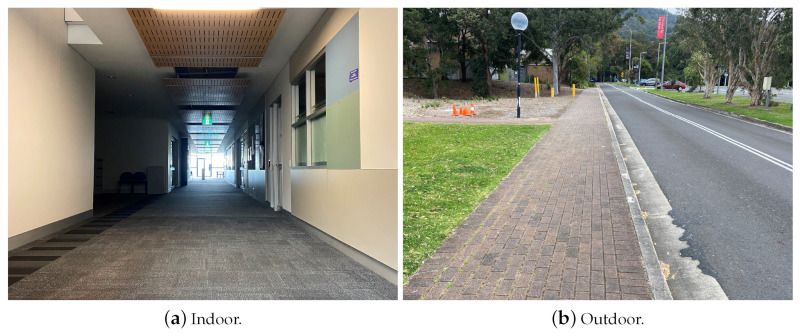
Experimental environments.

The indoor experiments were conducted along a corridor in one of the university buildings, while the outdoor ones were performed on pavement next to a car park, which can be considered as an open area, as shown in Figure 3. To improve the data reliability, unlike our previous experiments, more data values were collected for the work in this paper. In the previous works [34,35], each dataset contained ten separate experiments. One experiment only recorded the RSSI of a one-way direction walking or jogging. In this section, we significantly increased the size of each dataset by prolonging the experimental procedure in terms of time and distance.

**Figure 4:**
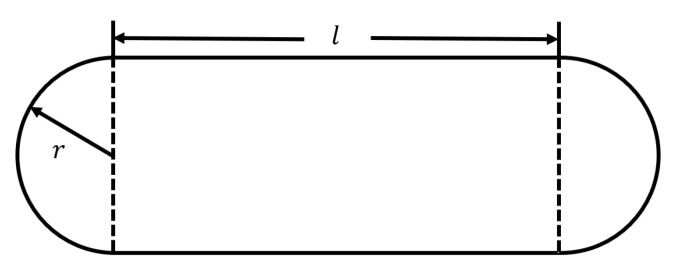
Schematic diagram of the experiment route.

With the purpose of making the path of the experiment identifiable and improving the reliability of the work, the route was traced with conspicuous cords stuck on the ground. As shown in Figure 4, the path had the shape of a rectangle, with two semi-circles on its left and right sides with a radius of r=0.96 m. The length of the long edge of the rectangle was l=32.53 m. The person under test was instructed to walk along the marked route. There were three remarks in the experimental procedure. First, in order to collect a dataset with continuous and abundant samples, the subject under test was required to walk along the path for twenty rounds in each experiment session. The total number of walking steps was counted to calculate the average walking step length $d_{0}$, which was used as a ground truth. Second, the participant performed the walk at a normal and steady pace at all times, especially during the arc areas, and did not make sharp turns at the semi-circle portions to ensure that the stride would not experience significant fluctuation. Third, new or newly charged rechargeable batteries were used before commencing a new set of experiments to guarantee the completeness of the data collection and the data reliability. Considering the time duration of each experiment and two AAA batteries powering the hardware, there might be excessive discharging of the two AAA batteries after each set of experiments.

## 4. System Model

As mentioned above, the transmitter and the receiver were fastened to the inner side of the ankles of the participant; thereby, an LoS path existed between the equipment throughout the time of the experiment. Moreover, no body tissue or other obstacles would appear between the transceivers; thus, the space between the two ankles could be regarded as a nearly free space. An experimental path loss model that measures the path loss between human ankles in daily activities was proposed in [35].

It could be described as a modified free space path loss model with a correction factor ΔPL:

(4) $$PL_{OA}\left(\mathrm{dB}\right)=PL_{FS}+\Delta PL,$$

where $PL_{FS}$ (dB) is the free space path loss and ΔPL (dB) is the correction factor, which consists of the hardware non-linearity, multipath propagation, insertion loss, and mismatch loss. From the observations in [35], the correction factor was empirically found as 10 dB. Therefore, Equation (4) can be written as

(5) $$PL_{OA}\left(\mathrm{dB}\right)=PL_{FS}+10.$$

It is noted that the free space path loss is numerically defined as

(6) $$PL_{FS}\left(\mathrm{dB}\right)=20\log_{10}\left(\frac{4\pi d}{\lambda }\right),$$

where *d* (m) is the distance between the two antennas and λ (m) is the signal wavelength. From Equations (5) and (6), this distance could be estimated as

(7) $$d=\frac{\lambda }{4\pi }10^{\left(\frac{PL_{OA}\left(\mathrm{dB}\right)-10}{20}\right)}.$$

Seen from the end device, the on-ankle path loss can be calculated as

(8) $$\begin{array}{cc} PL_{OA}\left(\mathrm{dB}\right) & =P_{t}-P_{r}+G_{t}+G_{r},\\ \; & =P_{t}+RSSI, \end{array}$$

where $P_{t}$ (dB) and $P_{r}$ (dB) are the transmitted power and the received power, respectively. Note that $P_{r}$ (dB) is a negative value. $G_{t}$ (dB) and $G_{r}$ (dB) are the antenna gains of the transmitter and the receiver. $RSSI=-P_{r}+G_{t}+G_{r}$, and the RSSI is presented as a positive decibel value from our experiments.

From Equations (7) and (8), the distance between the two transceivers is

(9) $$d=\frac{\lambda }{4\pi }10^{\left(\frac{P_{t}\left(\mathrm{dB}\right)+RSSI\left(\mathrm{dB}\right)-10}{20}\right)}.$$

To improve the accuracy of the step length estimation, a filtering technique was proposed in our paper [34] to remove path loss outliers by establishing a pair of path loss thresholds. Any path loss that is greater than the upper threshold or smaller than the lower threshold would be abandoned. The path loss values within the two thresholds were used to estimate the step length. The lower threshold was found to correspond to the point at which the survival rate of the dataset dropped to 0.68 [34]. The upper threshold was found from the second hump of the probability density function (PDF) of the measured data [34]. This hump could be approximated as a Gaussian distribution:

(10) $$f_{2}\left(x\right)=a_{2}e^{-{\left(\frac{x-b_{2}}{c_{2}}\right)}^{2}},$$

where $a_{2}$, $b_{2}$, and $c_{2}$ are all curve-fitting coefficients, whose values could be estimated by mathematical software, such as Matlab. $a_{2}$ is the amplitude and $b_{2}$ the centroid, while $c_{2}$ relates to the peak width of the Gaussian distribution. These coefficients have a relation with the mean μ and the standard deviation σ of the second hump based on the above Gaussian distribution in Equation (10) as follows:

(11) $$\mu =b_{2},$$

(12) $$\sigma =\frac{c_{2}}{\sqrt{2}}.$$

As mentioned in [34], the corresponding upper threshold was found as μ+γσ, where γ is the key factor in specifying the upper threshold.

The aim of this paper was to estimate the walking step length and update the estimation after each certain time period. This time period is the duration of each sliding time window. Thus, the collected RSSI dataset was truncated into different time windows $tw_{i},\left(i=1,2,...,k\right)$. The upper threshold of each time window $tw_{i}$ is determined as

(13) $$Th_{i}^{\left(u\right)}=\mu_{i}+\gamma \sigma_{i}.$$

Meanwhile, the lower threshold is fully dependent on the current time window sample:

(14) $$Th_{i}^{\left(l\right)}=Th_{sample}^{\left(l\right)},$$

where $Th_{sample}^{\left(l\right)}$ is the lower threshold value calculated for the current time window. $Th_{sample}^{\left(l\right)}$ is determined at the point where the survival rate of the data in this time window falls to 0.68. We propose here an EWMA algorithm, where the mean $\mu_{i}$ and standard deviation $\sigma_{i}$ in Equation (13) for the time window $tw_{i}$ are calculated recursively as

(15) $$\mu_{i}=\left\{\begin{array}{cc} \mu_{sample}, & i=1\\ \alpha \mu_{i-1}+\left(1-\alpha \right)\mu_{sample}, & i\geq 2 \end{array}\right.$$

(16) $$\sigma_{i}=\left\{\begin{array}{ccc} \sigma_{sample}, & \; & i=1\\ \beta \sigma_{i-1}+\left(1-\beta \right)\sigma_{sample}, & \; & i\geq 2 \end{array}\right.$$

where the subscript sample indicates the corresponding value for the current time window. In the following analyses, the influence of γ will be explored in different environments.

## 5. Experimental Results

In this section, human daily walking activities are examined in both indoor and outdoor environments. The datasets of the path loss values were collected to estimate the participant’s walking step length in both scenarios. Besides, the influence of the key parameter, γ, on the upper threshold in Equation (13) was investigated, assuming that the weights α and β in Equations (15) and (16) are α=0.125 and β=0.25, respectively. The values α=0.125 and β=0.25 were chosen for illustration purposes. They had small values to have more impact on the current sample values of μ and σ, denoted as $\mu_{sample}$ and $\sigma_{sample}$, on the average values of μ and σ. The rationale was that the current data samples were more significant than the old samples in the human step length estimation. Recall that $\mu_{sample}$ and $\sigma_{sample}$ are the mean and standard deviation, respectively, which could be found from the second Gaussian hump of the current time window. Research on the optimal pair of α and β in step length estimations is beyond the scope of this paper.

In each indoor or outdoor scenario, two datasets were collected for the completeness of the experiments. Hence, there were four datasets in total, and the real walking step length of each dataset is shown in Table 1. It is noted that the average walking step length for men and women is 2.5 feet and 2.2 feet, respectively [38]. In reality, step length may be impacted by several factors, such as height, age, injury, illness, terrain, and the environment surrounding the subject under test. Generally, the walking step length for adults is within the range of 67.1 cm to 76.2 cm on average [38]. As a result, in this paper, our measurements and analyses focused on the range of step length from 0.6 m to 0.8 m. In Table 1, the range of the real walking step length of the person under test was from 0.66 m to 0.68 m, which lies in our range of interest. In addition, in the same experimental scenario, during both experiments, the person under test walked along the pre-defined route at a normal speed for twenty rounds to collect sufficient data samples. Though the measurements were carried out in the same scenarios, there was still a slight discrepancy of the total walking time in different measurement trials. Therefore, the number of collected data samples varied, resulting in the different numbers of time windows among different experiments. Considering a time window size of 60 s, corresponding to 3000 samples per time window, each of these datasets had 11 to 15 time windows. It is noted that, due to the slight difference of total walking time and the difference of the hardware synchronisation time, the number of collected data samples, thus the number of time windows, may vary among different experiments. Moreover, compared to the experimental datasets in [34], the datasets in this paper contained twice the amount of data.

The benefit of increasing the size of the datasets is twofold. Firstly, it overcomes the limitation of the data samples in the previous experiments (around 15,000 samples in each experimental scenario), which may not be sufficient for applying the sliding time window and the EWMA. Secondly, the reliability of the collected data is likely to be improved with a more extensive dataset.

### 5.1. Indoor Walking

In our previous study [34], the histogram of the on-ankle path loss can be well approximated by a two-term Gaussian fitting curve model, whose second hump is closely related to the maximum path loss, which helps estimate the step length. An innovative filtering technique was proposed in [34] to filter out the path loss outliers by setting a pair of thresholds, namely the lower and upper thresholds. Recalling from our previous work in [34], the lower threshold could be numerically found as the corresponding path loss value when the survival rate of the dataset reaches 0.68. Meanwhile, the upper threshold is located on the right side of the second hump, and its value is a function of the mean and standard deviation of the second Gaussian hump, which can be expressed as

(17) $$Th^{\left(u\right)}=\mu +\sigma ,$$

which indicates γ=1 in [34]. In this paper, along with the proposed EWMA algorithm, we also investigated the optimal range of γ to obtain a more accurate step length estimation following Equation (13).

**Figure 5:**
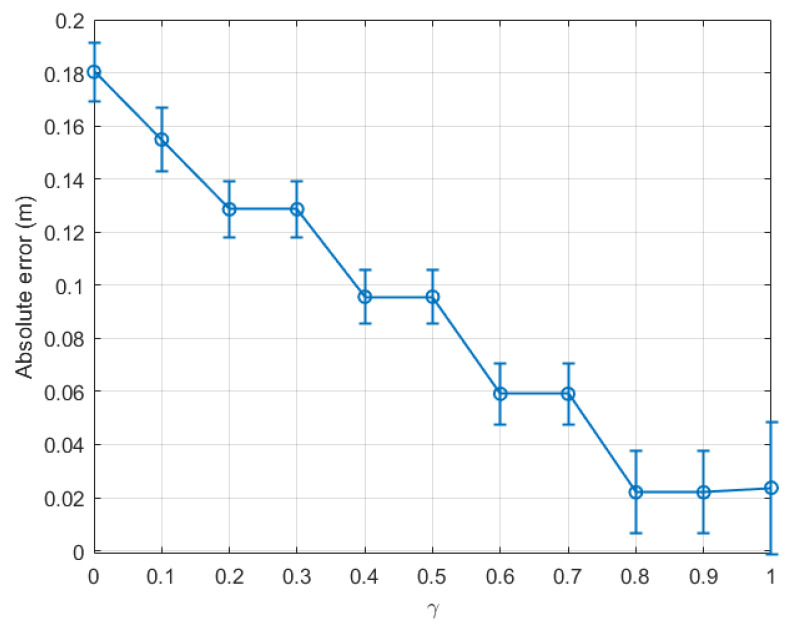
Indoor walking (dataset 1): absolute error of the last time window ($tw_{12})$ with respect to γ∈[0,1].

Figure 5 compares the absolute error between the real step length and the averaged estimation, calculated for the last time window ($tw_{12}$), with respect to the variation of γ from 0 to 1 for the indoor walking case based on dataset 1. The marker on each error bar in Figure 5 represents the average estimated absolute error, while the two caps present the variation of the estimated absolute error around the average value, i.e., the deviation of the estimation. The result showed that, when only the last time window $tw_{12}$ was considered, the absolute error (AE) dropped constantly from 0.18 m at γ=0 to nearly 0.02 m at γ=0.8. This error remained stable at γ=0.9 and then slightly rose at γ=1.

**Figure 6:**
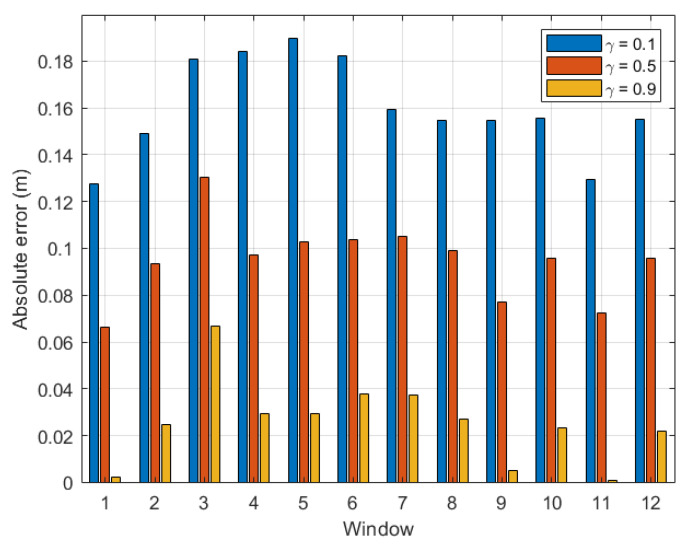
Indoor walking (dataset 1): absolute error with respect to time windows $tw_{i},i\in \left[1,12\right]$.

For more comprehension, Figure 6 plots the absolute error calculated for each time window with γ=0.1, 0.5, and 0.9 in a bar diagram. The same tendency as shown in Figure 5 can be seen in Figure 6. For instance, at the last time window, $AE_{\left(\gamma =0.1\right)}^{\left(tw_{12}\right)}>AE_{\left(\gamma =0.5\right)}^{\left(tw_{12}\right)}>AE_{\left(\gamma =0.9\right)}^{\left(tw_{12}\right)}$.

**Figure 7:**
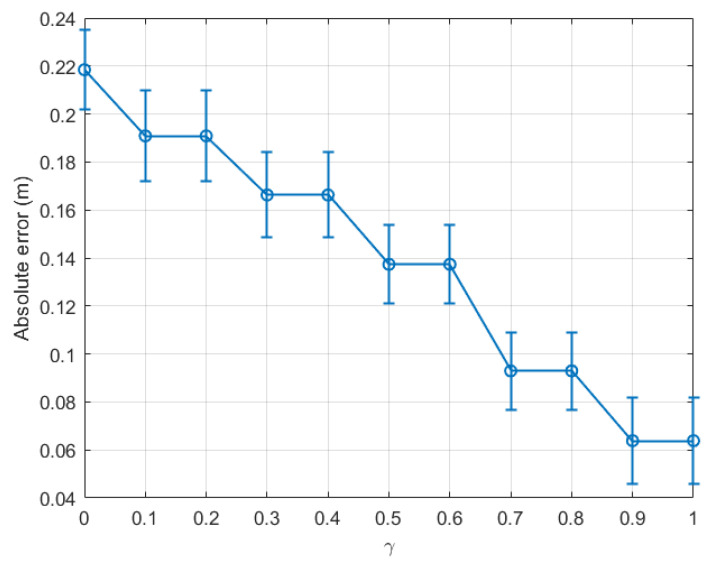
Indoor walking (dataset 2): absolute error of the last time window ($tw_{11})$ with respect to γ∈[0,1].

**Figure 8:**
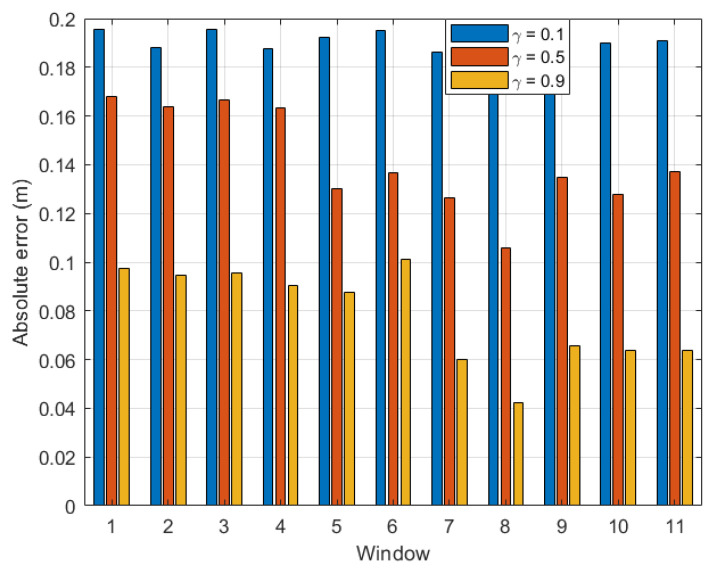
Indoor walking (dataset 2): absolute error with respect to time windows $tw_{i},i\in \left[1,11\right]$.

From the above experiment, it is clear that the optimal γ was located between 0.8 and 0.9 for indoor environments. For completeness, we plot a graph similar to Figure 5 for dataset 2 for the same indoor walking scenario as shown in Figure 7. Clearly, the absolute error calculated for the last time window decreased consistently with γ, and the optimal γ was within the range [0.9, 1.0]. Further, it is confirmed in Figure 8 that the optimal γ of this dataset was 0.9, and the absolute error could be as small as 0.064 m.

As a result, the optimal γ for calculating the upper threshold for an indoor walking activity in Equation (13) was found to be 0.9, which is relatively close to the sub-optimal value γ=1 chosen in [34]. The above estimation error could be as small as 3.02% of the real step length.

### 5.2. Outdoor Walking

Regarding the outdoor case, it was predicted based on [34] that the optimal γ could be around 0.5, because the multipath effect is less serious than the indoor case. This was confirmed by our experiments as detailed below.

**Figure 9:**
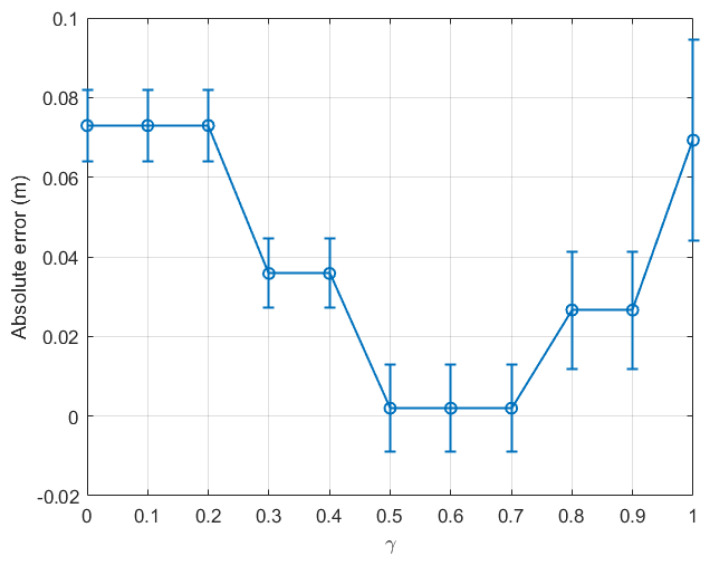
Outdoor walking (dataset 3): absolute error of the last time window ($tw_{15})$ with respect to γ∈[0,1].

**Figure 10:**
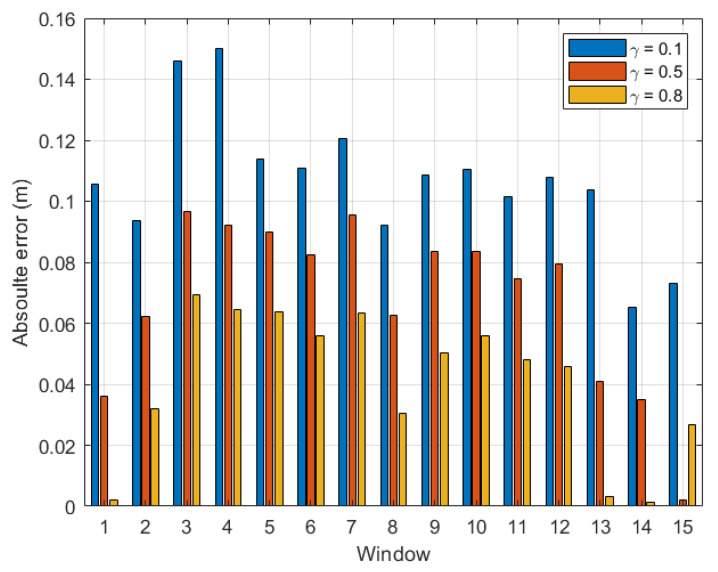
Outdoor walking (dataset 3): absolute error with respect to time windows $tw_{i},i\in \left[1,15\right]$.

Figure 9 presents the absolute error of the step length estimation with respect to γ for dataset 3. From Figure 9, at the last time window $tw_{15}$ of dataset 3, it was observed that the estimated absolute error reduced to only 0.002 m when γ was within the range [0.5, 0.7]. Figure 10 depicts the absolute error of the step length estimation with respect to the sliding time windows for dataset 3. From this figure, it is clear that the best γ for the outdoor walking scenario was around 0.5, which turned out to be the same value chosen in [34].

**Figure 11:**
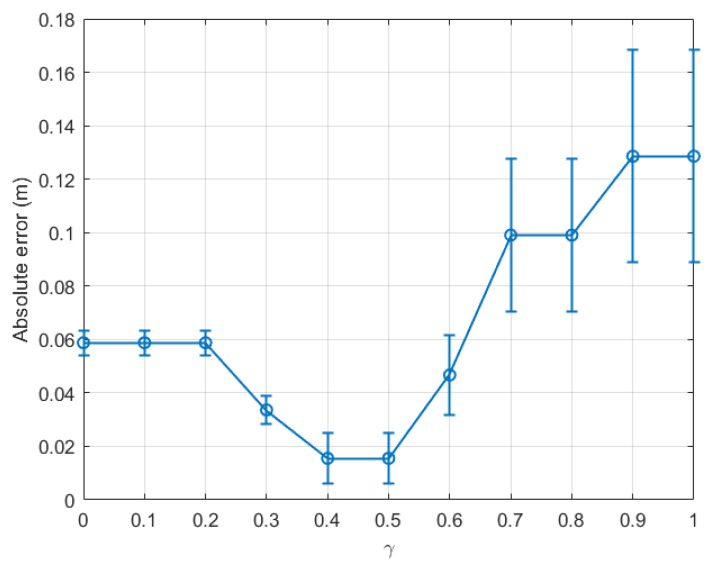
Outdoor walking (dataset 4): absolute error of the last time window ($tw_{11})$ with respect to γ∈[0,1].

**Figure 12:**
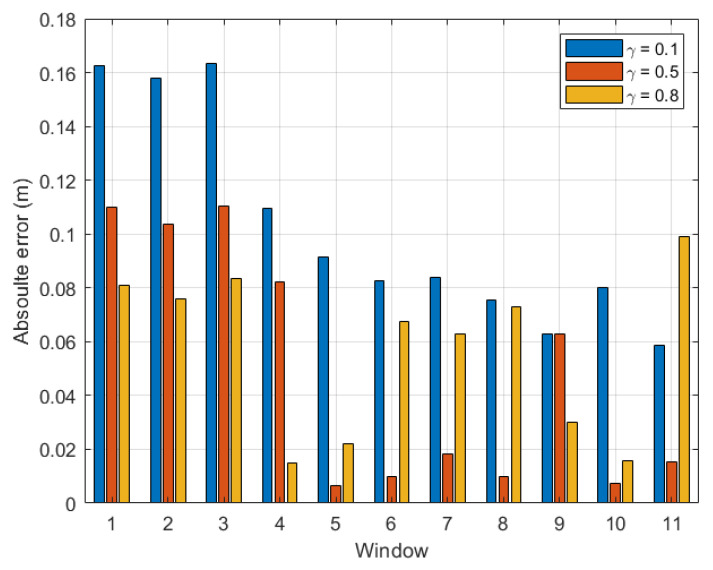
Outdoor walking (dataset 4): absolute error with respect to time windows $tw_{i},i\in \left[1,11\right]$.

For completeness, Figure 11 and Figure 12 present the estimation errors of another dataset of outdoor experiments, namely dataset 4. From Figure 11, it is noticed that the line graph shares the same trend as that for dataset 3 as presented in Figure 9. From the two figures, the smallest absolute error of the last time window $tw_{11}$ was 0.0155 m when γ was within the range [0.4, 0.5]. The step length estimation error for the outdoor scenario could be as small as 0.30% of the real step length.

**Figure 13:**
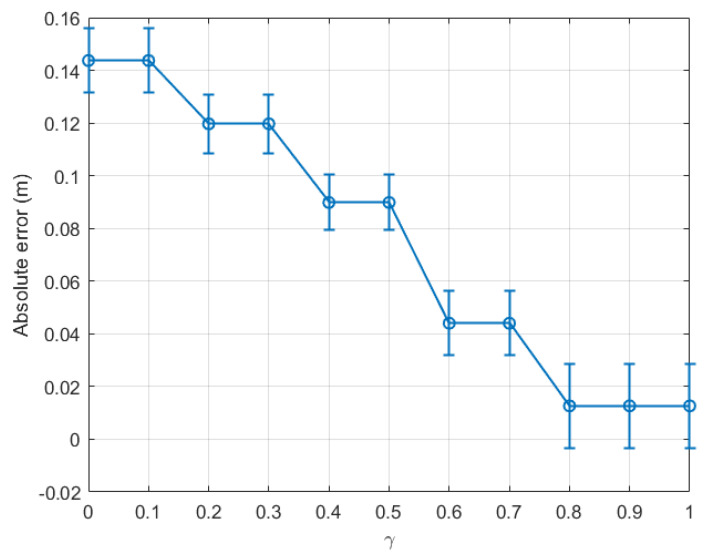
Indoor walking (dataset 5): absolute error of the last time window ($tw_{18})$ with respect to γ∈[0,1].

**Figure 14:**
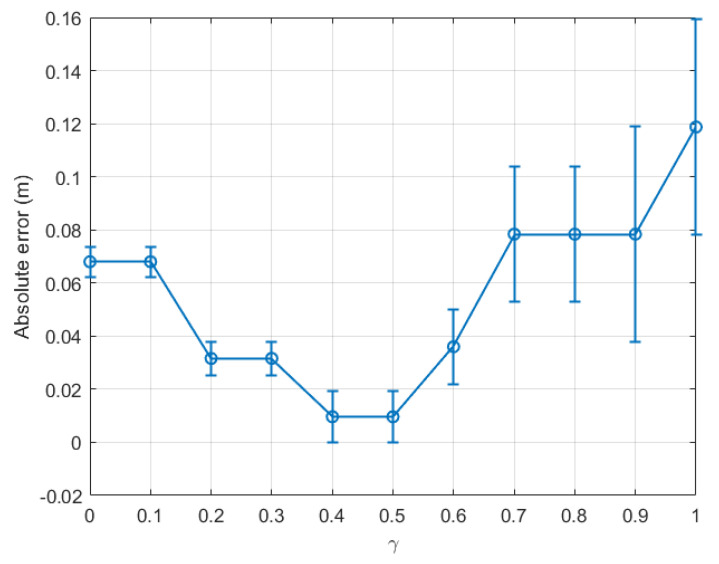
Outdoor walking (dataset 6): absolute error of the last time window ($tw_{18})$ with respect to γ∈[0,1].

In order to confirm the validity, accuracy, repeatability, and reliability of our proposed method, two more experiments were conducted to collect bigger datasets than the four datasets 1–4 mentioned previously in our analysis, for indoor and outdoor walking scenarios, respectively. The two newly collected datasets, namely datasets 5 and 6, contained 18 time windows each. The absolute step length measurement errors calculated based on the last time window $tw_{18}$ for datasets 5 and 6 are shown in Figure 13 and Figure 14 for the indoor and outdoor walking scenarios, respectively.

Figure 13 and Figure 14 show that the new results agree with those mentioned previously in the paper. Let us consider Figure 13 for illustration. The real step length for dataset 5 is 0.6679 m. As shown in this figure, when γ varied, the smallest absolute step length measurement error of 0.0127 m, i.e., 1.90% of the real step length, could be achieved when γ was in the range [0.8, 1]. Similarly, in Figure 14, the smallest absolute measurement error of 0.0096 m, i.e., 1.45% of the actual step length (0.6599 m), could be achieved when γ was in the range [0.4, 0.5]. These optimal γ ranges and the centimetre-level accuracy are similar to those mentioned previously for datasets 1–4.

To sum up, from the six groups of experiments, it was possible to achieve a centimetre-scale absolute error in step length estimation for both indoor and outdoor walking scenarios. Furthermore, the estimated absolute error of the outdoor walking case could be as small as a few millimetres (cf. Figure 9, Figure 10, and Figure 14). In general, the step length estimated in the outdoor walking scenario was more accurate than in the indoor walking one, as shown in Table 2. For instance, for indoor walking, from datasets 1, 2, and 5, the smallest absolute estimation error between the real step length and corresponding estimated step length was 0.0127 m. Meanwhile, for outdoor walking, the largest absolute step length estimation error based on the datasets 3, 4, and 6 was just 0.0155 m. It was observed that the estimated step length had a centimetre-level error for indoor scenarios, whereas it had a sub-centimetre-level error for outdoor ones. This observation agrees with the finding in our previous work in [34], where the SMA method was applied and the absolute error was 10.15 mm and 4.40 mm for indoor and outdoor walking, respectively. The comparative results validated the hypothesis proposed in this paper, that the proposed EWMA algorithm could be employed to update the upper threshold periodically. The estimated step length within each time window was estimated based on the updated pair of thresholds. The estimation accuracy was slightly worse than the experimental outcomes in [34] because the data being used in each time window were downsized. However, the proposed EWMA algorithm is promising because it allows real-time step length estimation without sacrificing much accuracy.

Recall that the work in [28] using a two-camera system had step length estimation outcomes with an error of 6%. The estimation error in the IR thermography technique is 9–22% [29]. By using the IMU sensor, the step length estimation error in the work [30] was about 3%, while the work [33] mounted the wearable sensor on the wrists of the subject under test, gaining an estimation error of 4.5%. From Table 2, the median relative estimation errors for indoor and outdoor walking scenarios in our EWMA technique were 3.12% and 1.45%, respectively. Compared with the existing techniques, the proposed EWMA algorithm could achieve a comparable or even better accuracy level in the step length estimation, while overcoming some main limitations of the existing techniques, which are discussed in Section 1.

### 5.3. Comparison of the Processing Time

Apart from the accuracy evaluation, the processing time, which is the time used to estimate the step length, was analysed to assess the effectiveness and the feasibility of the proposed EWMA algorithm. In this section, we compare the processing time of our measurement technique in the cases of with and without applying the proposed EWMA algorithm. It is noted that the measurement technique without applying the EWMA algorithm is the technique proposed in [34]. For a fair comparison, we used datasets of the same sizes. The datasets were those for the indoor and outdoor walking experiments in [34], which had 6 time windows and 4 time windows, respectively. Each time window comprises 3000 data samples. The processing time to output a value of the step length depends on the number of samples of the datasets to be used each time and whether the EWMA algorithm is used. The measurement technique in [34] requires the use of the whole dataset to estimate the step length; thus, the processing time is large. Meanwhile, the processing time is significantly reduced in the EWMA technique due to the shortened time window.

**Figure 15:**
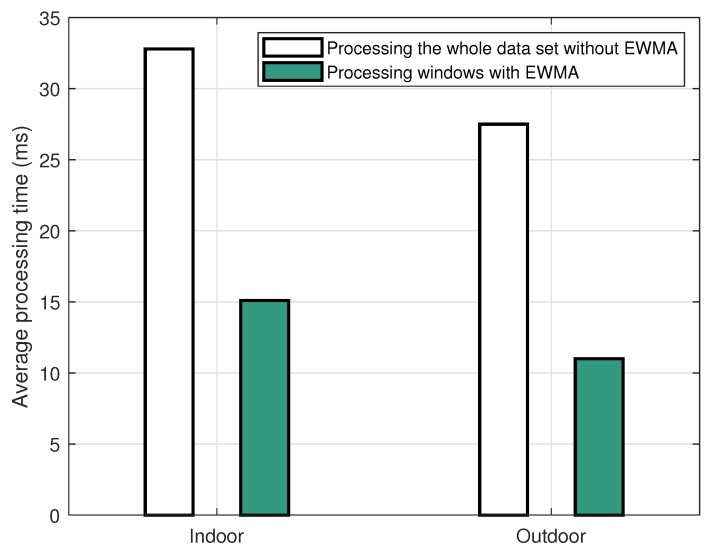
Comparison of the average processing time in the cases with and without the EWMA, for indoor and outdoor walking scenarios.

The above conjecture is confirmed in Figure 15, which plots the average processing time of step length estimation for the same dataset with and without the proposed EWMA algorithm. In this figure, the white bars are the time required to estimate the step length using the whole dataset without applying the EWMA algorithm in indoor and outdoor activities. The green bars are the average time of step length estimation required by the proposed EWMA algorithm to process each time window. This figure shows that the processing time was significantly shortened by the EWMA algorithm for both indoor and outdoor scenarios. Initially, the indoor walking case included more data samples than the outdoor one; thus, the time used to estimate the step length for indoor walking would be longer for outdoor walking without applying the proposed EWMA algorithm. However, with the application of EWMA algorithm, the step length could be estimated and updated in each time window. Based on the data samples collected from our previous work [34], the average processing time to estimate a step length was 15.1 ms and 11.0 ms, respectively. In particular, in the indoor walking scenario, the time required to estimate the step length in each time window using the EWMA algorithm was only 46.04% of the total time required for the step length estimation where the EWMA algorithm was not applied. For the outdoor walking case, this number was 40%. In other words, the proposed EWMA algorithm not only can provide comparable accuracy, but also shorten the processing time by 53.96% for indoor walking and by 60% for outdoor walking, compared to our technique proposed in [34].

## 6. Conclusions

In this paper, we estimated the human step length for daily indoor and outdoor walking activities based on our developed wearable transceivers and the RSSI method. The RSSI outlier filtering technique was refined with the proposed EWMA method, so that the thresholds could be updated periodically while the person under test was walking. As a result, the proposed EWMA algorithm facilitates the step length estimation in real-time. In addition, this paper investigated the optimal γ value in the equation of the upper threshold $Th^{\left(u\right)}$ to minimise the estimation errors, by analysing the statistical properties of the collected datasets. The results revealed that the optimal γ for an indoor environment was about 0.9, while this value for an outdoor scenario was around 0.5. With the optimal values of γ, step length estimation can be considered to be accurate with the estimation errors being as small as 3.02% and 0.30% for the indoor and outdoor scenarios, respectively. It is worth noting that this accuracy was achievable without the need to collect and process the whole dataset, as discussed in [34]. In addition, by comparing the processing time required to compute and output a step length estimation for the cases with and without using the proposed EWMA algorithm, it was found that the new method could shorten this processing time by 53.96% for indoor walking and by 56.72% to 60% for outdoor walking, respectively. As a result, the EWMA algorithm could be a promising candidate technique for measuring the step length in human daily activities in real-time.

## Figures and Tables

**Table 1:** Real walking step length and the number of time windows in the datasets.

Dataset	Description	Real Walking Step Length (m)	Number of Time Windows
1	Indoor walking 1	0.6627	12
2	Indoor walking 2	0.6844	11
3	Outdoor walking 1	0.6633	15
4	Outdoor walking 2	0.6561	11

**Table 2:** Comparison of the real step length, estimated step length, and absolute and relative estimation error for both indoor and outdoor walking environments.

Environment	Dataset	Real Step Length (m)	Estimated Step Length (m)	Absolute Estimation Error (m)	Relative Estimation Error (%)
Indoor	1	0.6627	0.6427	0.0200	3.12
	2	0.6844	0.6204	0.0640	9.35
	5	0.6679	0.6552	0.0127	1.90
Outdoor	3	0.6633	0.6613	0.0020	0.30
	4	0.6561	0.6409	0.0155	2.36
	6	0.6599	0.6503	0.0096	1.15

## Data Availability

The data presented in this study are available upon request from the corresponding author.

## Abbreviations

The following abbreviations are used in this manuscript:

AE	Absolute error
EMA/EWMA	Exponential (weighted) moving average
GPS	Global Positioning System
IDE	Integrated Development Environment
IMU	Inertial measurement unit
IR	Infrared radiation
ISM	Industrial, Scientific, and Medical
LoS	Line of sight
PDF	Probability density function
RF	Radio frequency
RSSI	Received signal strength indicator
SMA	Simple moving average
WMA	Weighted moving average
X-CTU	XBee Configuration & Test Unit
